# Role and function of *Chondrostereum purpureum* in biocontrol of trees

**DOI:** 10.1007/s00253-020-11053-5

**Published:** 2020-12-19

**Authors:** Leena Hamberg, Timo Saksa, Jarkko Hantula

**Affiliations:** 1 Natural Resources Institute Finland, P.O. Box 2, (Latokartanonkaari 9), FI-00790 Helsinki, Finland; 2 Natural Resources Institute Finland, Juntintie 154, FI-77600 Suonenjoki, Finland

**Keywords:** Forestry, Sprout control, Decay fungus, Stump mortality

## Abstract

**Abstract:**

A decay fungus, *Chondrostereum purpureum* (Pers. Ex Fr.) Pouzar, has been investigated in Europe, Northern America and New Zealand for its ability to decay hardwood stumps and thus prevent sprouting. The aim of these investigations has been to find an alternative to mechanical (cutting only) and chemical sprout control (cutting and applying chemicals to stumps in order to prevent sprouting). Mechanical sprout control is not an efficient option due to hardwood tree species’ ability to re-sprout efficiently after cutting, and therefore management costs are high. Chemicals would be efficient but due to their harmful effects on the environment, alternatives are needed. The fungal treatment, i.e., cutting accompanied with *C. purpureum* inoculum is an environmentally friendly and efficient option for sprout control. This mini-review comprises the role and function of *C. purpureum* in biocontrol of trees: the ecology of *C. purpureum*, its sprout control efficacy, factors affecting sprout control efficacy, devices in biological sprout control, potential risks, and the future perspectives of biological sprout control.

**Key points:**

*• A fungus Chondrostereum purpureum is efficient in preventing sprouting of hardwoods*

*• C. purpureum is not sensitive to environmental conditions*

*• Devices should be developed for cost-efficient biological sprout control*

## Introduction

Microorganisms such as fungi and bacteria, or the compounds that they produce, have potential as biological control agents in preventing the growth of harmful pests and diseases as described in many mini-reviews (Keswani et al. 2019; Memariani and Memariani 2020; Mukherjee et al. 2020; Tong and Feng 2020; Torracchi et al. 2020). Furthermore, this approach could also be utilized in restricting the growth of unwanted hardwood tree species (de Jong 2000; Wall 1990), for example in forest management, under electric power lines, above gas pipelines and next to roads and railways (de la Bastide et al. 2002; Hamberg et al. 2015).

In forestry, specific tree species are cultivated to produce timber and biomass for industry. Usually, the desired tree saplings must compete with naturally grown tree species for growing space, solar radiation, water and nutrients, and therefore, unwanted saplings are removed to promote the growth of more valuable cultivated trees (Huuskonen and Hynynen 2006; Huuskonen et al. 2020; Wagner et al. 2006). However, unwanted trees are often hardwoods that can sprout efficiently from stumps (Becker et al. 2005; Hamberg et al. 2020; Jobidon 1998; Lygis et al. 2012; Mallik et al. 2002; Wall 1990), and therefore additional pre-commercial thinning (PCT) operations are usually needed (Äijälä et al. 2019; Thiffault and Roy 2011).

Since hardwood thickets often need to be removed repeatedly, the cost of sprout control increases considerably (de la Bastide et al. 2002). An efficient option to control excessive growth of hardwood thickets would be chemicals (Bellgard et al. 2014; Harper et al. 1999; Lygis et al. 2012; Pitt et al. 1999) but public concern and the harmful effects of chemicals on the environment have restricted their use (de la Bastide et al. 2002; Bellgard et al. 2014; Benachour and Séralini 2009; Mallik et al. 2002; Dumas et al. 1997; Graymore et al. 2001; Lygis et al. 2012; Thiffault and Roy 2011; Vandenbroucke et al. 2005; Wagner 1993; Wagner et al. 2006; Wall 1990).

An environmentally friendly option to prevent hardwood sprouting is a decay fungus, *Chondrostereum purpureum* (Pers. Ex Fr.) Pouzar, that can be utilized as a biological control agent due to its efficient ability at preventing sprouting, potentially even as efficiently as chemicals (Becker et al. 2005; Bellgard et al. 2014; Harper et al. 1999; Jobidon 1998; Lygis et al. 2012). Consequently, the use of this fungal species as a biocontrol agent against weed hardwoods has been investigated in the Netherlands, Belgium, Lithuania, Finland, Canada, and New Zealand (Becker et al. 2005; Bellgard et al. 2014; de Jong 2000; Dumas et al. 1997; Hamberg et al. 2015, 2018, 2020; Lygis et al. 2012; Roy et al. 2010; Spiers and Hopcroft 1988; van den Meersschaut and Lust 1997; Vartiamäki et al. 2008a). In this mini-review, the role and function of *C. purpureum* in biocontrol of trees is described.

### Ecology of a decay fungus *Chondrostereum purpureum*

**Fig. 1:**
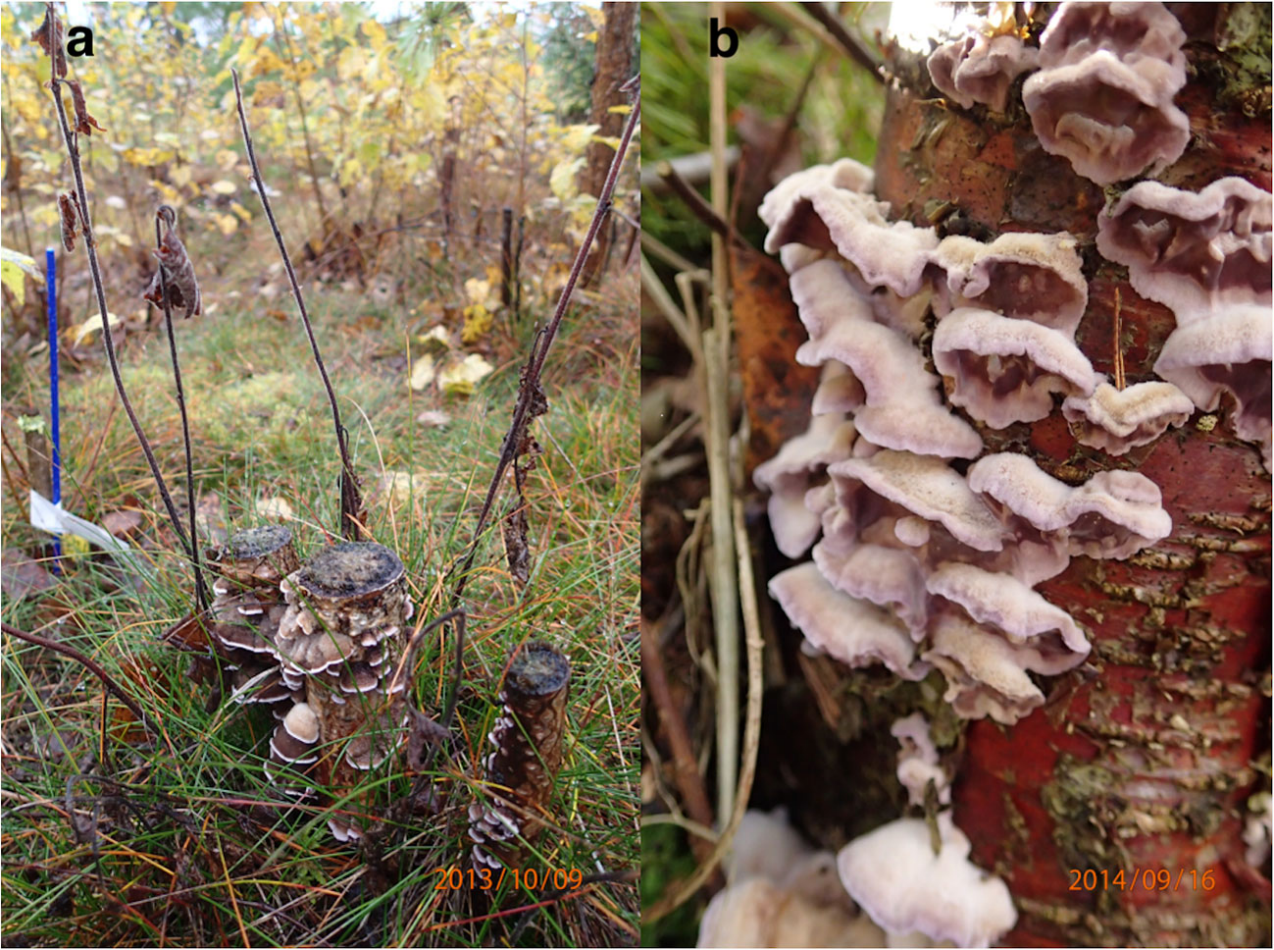
**a** Fruiting bodies of a decay fungus *Chondrostereum purpureum* in birch (*Betula pendula* Roth and *B. pubescens* Ehrh.), and stump sprouts killed by the fungus. **b** The basidiocarps of this fungus are purple. Photographs: Leena Hamberg

*C. purpureum* is a saprophytic or a weakly pathogenic pioneer species invading mainly stumps, branches, and trunks of deciduous tree species, but it is sometimes detected also in conifers as a saprophyte (Etheridge and Morin 1963; Gosselin et al. 1995; Ramsfield et al. 1996). The basidiocarps of this fungus are purple and often found on stumps and dead wood remains of broadleaved trees (Fig. 1). The fungus can also cause silver leaf disease in fruit trees (Percival 1902).

As a typical basidiomycete, the spore release of *C. purpureum* depends on high humidity (Spiers and Hopcroft 1988). It is capable of infecting trees only through open vessels in fresh wounds in branches, stems, or roots (Becker et al. 2005; Brooks and Moore 1923; Butler and Jones 1949; de Jong 2000; Erikson and Ryvarden 1973; Gosselin et al. 1999; Hamberg et al. 2017). The fungus grows through xylem tissues of the host plant and blocks vessels, causes cambial necrosis, decay, sapwood staining, and often death of the host (Rayner 1977; Wall 1986, 1991). The mechanism of its action is not fully understood, but it is associated with toxic polygalacturonases and laccases, as well as lignin- and manganese peroxidase activities (Miyairi et al. 1977; ten Have and Teunissen 2001; Vartiamäki et al. 2008a).

*C. purpureum* occurs commonly in boreal and temperate vegetation zones and is also present in New Zealand as an introduced species (Ramsfield et al. 1996). The populations between Europe and North America belong to the same biological species, but molecular markers show certain levels of differentiation between the continents, while intracontinental population differences are small (Becker et al. 2004, 2005; Gosselin et al. 1996, 1999; Hamberg et al. 2018; Ramsfield et al. 1996, 1999; Vartiamäki et al. 2008b).

### Sprout control efficacy

The sprout control efficacy of *C. purpureum* is based on its efficiency in disrupting the physiology of a treated tree and finally killing it. In biological sprout control, hardwood trees are cut and an inoculum medium—containing the hyphae of *C. purpureum* diluted with water or formulated as a gel-based paste—is spread or laid immediately on freshly cut stump surfaces (Bellgard et al. 2014; de Jong 2000; Hamberg et al. 2015). When inoculum medium is spread on freshly cut stump surfaces, *C. purpureum* gains an advantage over other naturally occurring decay fungi (Brooks and Moore 1926), penetrates the stumps, starts to decay wood, and finally kills the host when living tissues have been killed and decayed sufficiently (Hamberg et al. 2017). Decaying wood is a biological process and therefore treated stumps are not killed immediately. Depending on the ability of a fungal strain to decay wood and the resistance of a host tree, stump mortality begins ca. 2 months later and continues for up to at least 4 years after the treatment (Hamberg et al. 2014, 2015, 2017; Lygis et al. 2012, Table 1).

**Table 1:** Stump mortalities after the *Chondrostereum purpureum* treatments and control (cutting only or cutting only with inoculum medium without fungal hyphae) in different hardwood species. Values for the best fungal strain or for optimal application time are provided in case several strains or application times were investigated. Stump mean diameter and/or diameter range has been provided when available

Tree species	Scientific name	Stump diameter (cm)	Stump mortality (%)		Reference
			Fungal treatment	Control	
Box elder	*Acer negundo*	3.5 (1–12)	95^b^	33	Lygis et al. 2012
Gray alder	*Alnus incana*	3.5 (1–12)	65^b^	41	Lygis et al. 2012
		8.3 (0.6–24.3)	91^b^	43	Hamberg and Hantula 2018
Red alder	*Alnus rubra*	5–10	100^b^	86	Becker et al. 2005
Sitka alder	*Alnus viridis ssp. sinuata*	2.2	90^c!^	11	Harper et al. 1999
Paper birch	*Betula papyrifera*	5.4	99^c^	56	Jobidon 1998
Silver birch	*Betula pendula*	3.5 (1–12)	100^b^	20	Lygis et al. 2012
Silver and downy birch	*Betula pendula* and *B. pubescens*	3.5	92^b^	57	Vartiamäki et al. 2008a
		3.0 (2.1–4.4)	96^c^	60	Vartiamäki et al. 2009a
		1.1	78^c^	9	Hamberg et al. 2015
		1.4	86^c^	17	Hamberg et al. 2020
		11.2 (1.0–30.8)	97^b^	56	Hamberg and Hantula 2018
Largetooth and trembling aspen	*Populus grandidentata* and *P. tremuloides*	5–60	37^a-b^	12	Dumas et al. 1997
Poplar	*Populus euramericana*	40–60	100^b^	15	de Jong 2000
European aspen	*Populus tremula*	1.9	77^d^	52	Hamberg et al. 2014
		1.2	78^c^	47	Hamberg and Hantula 2016
		3.5 (1–12)	100^b^	75	Lygis et al. 2012
Trembling aspen	*Populus tremuloides*	–	84^c^	31	Harper et al. 1999
Black cherry	*Prunus serotina*	–	95^b^	–	de Jong 2000
		2–6	99^b^	53	Scheepens and Hoogerbrugge 1989
		–	91^b^	44	van den Meersschaut and Lust 1997
Pin cherry	*Prunus pensylvanica*	4.6	86^c^	35	Jobidon 1998
Black locust	*Robinia pseudoacacia*	3.5 (1–12)	7^b^	2	Lygis et al. 2012
Goat willow	*Salix caprea*	3.5 (1–12)	57^b^	23	Lygis et al. 2012
Rowan	*Sorbus aucuparia*	1.8	50^d^	14	Hamberg et al. 2014
Gorse	*Ulex europaeus*	1.1 (0.6–1.8)	66^!!^	50^!!^	Bourdôt et al. 2006

^a^Sprout control efficacy measured one growing season after the treatments

^b^Sprout control efficacy measured two growing seasons after the treatments

^c^Sprout control efficacy measured three growing seasons after the treatments

^d^Sprout control efficacy measured four growing seasons after the treatments

^!^Clump mortality

^!!^Mean values provided for different application times

The efficacy of different fungal strains of *C. purpureum* to kill treated stumps varies, with some strains more efficiently preventing sprouting than others (Bellgard et al. 2014; Hamberg and Hantula 2016; Hamberg et al. 2015; Harper et al. 1999; Jobidon 1998; Lygis et al. 2012; Pitt et al. 1999; Scheepens and Hoogerbrugge 1989; Vartiamäki et al. 2008a; Wall et al. 1996). The efficacy of different strains has been tested in laboratory conditions using different approaches. *C. purpureum* hyphae have been laid on Petri plates including wood tissue cultures or on tree cuttings, and the effect of a strain on tissue or cutting mortality then investigated (Bellgard et al. 2014; Eckramoddoulah et al. 1993; Spiers et al. 1998, 2000; Wall et al. 1996). In the laboratory, the laccase and manganese peroxidase enzyme production of a *C. purpureum* strain has been shown to correlate with its sprout control efficacy in the field, and therefore enzyme tests have been used in screening potential fungal strains (Hamberg et al. 2015; Vartiamäki et al. 2008a). Also, a short-term breeding process has been utilized to crossbreed efficient fungal strains (Hamberg et al. 2015). Pairing of mycelia for breeding is easy to perform in the laboratory. Two homokaryotic hyphae—both originating from a single spore of *C. purpureum*—are laid on the same growth substrate on a Petri plate, and when hypha meet on a plate they can form a new heterokaryotic *C. purpureum* strain, similarly as it happens on wood in nature (Butler and Jones 1949; Hamberg et al. 2015; Spiers et al. 2000; Wall et al. 1996). Thereafter, the best strain for biological control can be selected based on the efficacy of new strains in decaying stumps and killing host trees in field investigations or, e.g., efficacy-associated enzymatic activities (Hamberg et al. 2015).

Since the 1980s, the efficacy of different *C. purpureum* strains as biological control agents has been tested in several different hardwood trees (Hamberg and Hantula 2018; Hamberg et al. 2015; Lygis et al. 2012; Scheepens and Hoogerbrugge 1989; Vartiamäki et al. 2008a; Wall 1990, Table 1). *C. purpureum* has been especially efficient in controlling sprouting of birch (*Betula papyrifera* Marsh., *B. pendula*, *B. pubescens*), alder (*Alnus incana* (L.) Moench, *A. rubra* Bong.), box elder (*Acer negundo* L., 1753), and cherry species (*Prunus serotina* Ehrh., *P. pensylvanica* L. fil.) with stump mortalities of 80–100% after the treatment (Becker et al. 2005; Hamberg and Hantula 2018; Hamberg et al. 2015, 2020; Jobidon 1998; Lygis et al. 2012; Scheepens and Hoogerbrugge 1989), but promising results have been achieved also for aspen (*Populus tremula* L., *P. tremuloides* Michx.) (Hamberg and Hantula 2016; Hamberg et al. 2014; Harper et al. 1999; Lygis et al. 2012). Efficacy has been lower in rowan (*Sorbus aucuparia* L.), goat willow (*Salix caprea* L.), and black locust (*Robinia pseudoacacia* L.) with stump mortalities less than 60% (Hamberg et al. 2014; Lygis et al. 2012).

Although *C. purpureum* does not always kill its host, it may affect the number and height of living sprouts. Reduction in the number of stump sprouts or delay in the development of stump sprouts after the *C. purpureum* treatment, compared to control (cutting only), has been observed in birch (*Betula pendula* and *B. pubescens*) (Hamberg et al. 2015; Vartiamäki et al. 2009a). The height of living sprouts has been considerably lower after the *C. purpureum* treatment than cutting only, e.g., in birch (*Betula pendula* and *B. pubescens*), rowan (*Sorbus aucuparia*), and European aspen (*Populus tremula*) but also in other hardwoods (Hamberg n.d.; Hamberg et al. 2014; Vandenbroucke et al. 2005; Vartiamäki et al. 2008a). Height reduction is promising in terms of forest management, as desired tree species can more easily gain an advantage over unwanted hardwoods.

Some hardwood species, such as rowan (*Sorbus aucuparia*), aspen (*Populus tremula*, *P. tremuloides*), and gray alder (*Alnus incana*), also produce root suckers that increase the number of competing saplings around cultivated trees (Hamberg and Hantula 2018; Harper et al. 1999; Worrell 1995; Zerbe 2001). Rowan, aspen and gray alder are capable of allocating resources to root sucker production especially if stump sprouting is restricted due to fungal treatment with *C. purpureum* (Hamberg and Hantula 2018; Hamberg et al. 2011, 2014), and therefore with these tree species, sprout control is more difficult at the stand level (see e.g., Harper et al. 1999; Lygis et al. 2012). Currently, no long-term investigations have been performed to show how far the fungus can penetrate along underground stems and roots and for how long the treated stumps can produce root suckers after stumps have been decayed. However, living, aboveground stems belonging to the same clone as a treated stump can support suffering parts within the clone (Hamberg and Hantula 2016; Hamberg et al. 2014). Thus, cutting and applying *C. purpureum* inoculum for all unwanted saplings potentially belonging to the same clone is recommended in order to increase biocontrol success.

### Factors affecting sprout control efficacy

#### Host tree

The ability of *C. purpureum* to infect different tree species and saplings varies greatly due to the ability of a tree to resist fungal infection (see above), and the physiological state of a tree (Hamberg n.d.; Heide 2011; Stanislawek et al. 1987; Vartiamäki et al. 2009a; Wall 1990). Some evidence has been found that the most severe effects of the fungus take place when the amount of soluble carbohydrates is highest in wood, i.e., when a tree is growing intensively (Brooks and Moore 1926; Butler and Jones 1949; Lygis et al. 2012; Stanislawek et al. 1987). Thus, an important component in terms of sprout control efficacy is timing, i.e., applying fungal inoculum when the resistance of trees against *C. purpureum* is lowest.

Seasonal effects on the sprout control efficacy of *C. purpureum* have been found in several tree species. In birch (*Betula pendula* and *B. pubescens*), the sprout control efficacy of *C. purpureum* is good when the treatment is performed in spring or summer but it seems to decrease towards the end of the growing season (Hamberg et al. 2015, 2017; Laine et al. 2020a; Vartiamäki et al. 2009a). The reason for decreasing efficacy towards the end of a growing season is not fully understood. In several tree species, summer treatments have resulted in higher stump mortalities than those of autumn treatments (pooled data from *Betula pendula*, *Alnus incana*, *Populus tremula*, *Salix caprea* and *Acer negundo* in Lygis et al. 2012). In *Prunus* and *Salix*, susceptibility to *C. purpureum* is greatest in spring and early summer, and least in winter (Spiers et al. 1998). In *Prunus serotina* Ehrh., both spring and autumn were equally suitable for *C. purpureum* infection (Scheepens and Hoogerbrugge 1989). In rowan (*Sorbus aucuparia*), sprout control efficacy is highest in summer while in spring and autumn, it is clearly lower (Hamberg n.d.; Hamberg et al. 2017), probably because at that time most of the resources are in underground stems and roots (Heide 2011; Millard 1996; Millard et al. 2001), and thus available after cutting for regrowth and defending against fungal infection. This effect is largely regulated by temperature (Heide 2011). In aspen (*Populus tremula*), the fungal treatment has been successful early in spring (Hamberg and Hantula 2016), possibly because at that time carbohydrates are transported from underground parts to shoots (Johansson 1993). However, the amounts of defensive compounds in wood may also regulate the success of *C. purpureum* treatment during a growing season (Palo 1984).

Increasing stump diameter has been associated with increasing stump mortality after *C. purpureum* treatment (Salmi 2017). In a large stump, there is more space for a higher number of fungal fragments to colonize it and cause stump decay, leading to mortality sooner than in smaller stumps. When birch (*Betula pendula* and *B. pubescens*) saplings 0.5–6.0 cm in stump diameter were cut and treated with *C. purpureum* inoculum, larger stumps died faster than smaller stumps (Hamberg and Hantula 2020; Hamberg et al. 2020). However, when the diameter of experimentally treated stumps ranged from 0.6 to 30.0 cm, the results showed that the smallest and largest stumps were most susceptible to the fungal treatment, with trees ca. 13 cm in stump diameter being the most resistant, whereas in gray alder (*Alnus incana*), all stumps irrespective of stump diameter were prone to the treatment (Hamberg and Hantula 2018). After the second growing season, almost all treated stumps had died and no effect of basal diameter could be observed.

Wound susceptibility to *C. purpureum* infection decreases clearly with time (Brooks and Moore 1926; Spiers and Hopcroft 1988), and therefore fungal inoculum should be spread to stump surfaces immediately after a sapling has been cut. Even a 15–30 min delay between cutting and spreading of fungal inoculum decreases the sprout control efficacy of *C. purpureum* (Hamberg and Hantula 2020). Delay in spreading especially affects the treatment efficacy on stumps with small diameters. Observations in the field have shown that immediately after cutting, the fungal inoculum will be sucked into a stump but after a delay, it stays as a drop on the stump surface, probably due to the closing of the vessels.

#### Site-specific factors

After *C. purpureum* treatment, some site-specific effects on tree mortality have been observed (Hamberg et al. 2017; Pitt et al. 1999). Increasing soil moisture levels have been associated with an increase in stump mortality rates of birch (*Betula pendula* and *B. pubescens*), but this effect disappeared during the second growing season following stump treatment (Hamberg and Hantula 2020). Growing space affects the ability of *C. purpureum* to kill inoculated stumps. Both increasing volume of standing trees (> 5 cm in diameter at breast height) and the number of other saplings around a treated stump increase stump mortality (Hamberg et al. 2014, 2015). However, in clonal species, such as European aspen (*Populus tremula*), conspecific mature trees around an investigated stump—possibly belonging to the same clone—can provide support for stumps treated with *C. purpureum* (Hamberg and Hantula 2016).

#### Weather

Weather conditions affect *Chondrostereum purpureum*, although these effects are often temporal and milder than those of a host tree. In laboratory investigations, the optimum growth temperature for *C. purpureum* is 24–25 °C but the growth is severely inhibited at near zero and in temperatures over 35 °C (Eckramoddoulah et al. 1993; Hamberg and Hantula 2020; Spiers and Hopcroft 1988; Spiers et al. 2000; Stanislawek et al. 1987; Wall 1986). However, in the field, the mycelium can survive during winter, although its growth is limited due to low temperatures and low soluble carbohydrate concentration in the wood (Butler and Jones 1949; Scheepens and Hoogerbrugge 1989), and *C. purpureum* is able to withstand high temperatures (even 30–40 °C) in the field (Dumas et al. 1997; Hamberg and Hantula 2020). High temperatures may initially delay the sprout control efficacy of *C. purpureum* (i.e., the growth of mycelia within a stump) but two growing seasons after the fungal treatment, this effect on stump mortality is no longer observed (Hamberg and Hantula 2020).

Wood moisture content is an important factor determining decay (Boddy and Rayner 1983), and therefore *C. purpureum* benefits from optimal moisture conditions (Hamberg and Hantula 2020). If moisture content is high in living wood, it can limit fungal infection, but water shortage as a stress factor may make trees prone to infection (Boddy and Rayner 1983; Hamberg and Hantula 2020; Pitt et al. 1999). Thus, low precipitation before the *C. purpureum* treatment helps the fungus to penetrate the wood of a host suffering from water stress, while an increase in precipitation after the treatment provides suitable moisture conditions for the fungus to invade deeper into the wood (Hamberg and Hantula 2020). *C. purpureum* is not sensitive to heavy rains during stump treatments, although fungal inoculum may be easily washed from stump surfaces (Hamberg et al. 2020). In birch (*Betula pendula* and *B. pubescens*), rain showers may initially delay the sprout control efficacy of *C. purpureum* but three growing seasons after the fungal treatment, stump mortality is as high for stumps treated in a rainstorm as in sunny weather (Hamberg et al. 2020).

### Devices in biological sprout control

Most biological sprout control experiments have been performed by spraying or spreading *C. purpureum* inoculum on freshly cut stumps manually after a cutting operation (e.g., Hamberg et al. 2014; Roy et al. 2010). In practical forestry, pre-commercial thinning (PCT) is done with clearing saws or, in a small proportion, with fully mechanized devices fitted on forest machines. In these practical solutions, the spreading of the biocontrol agent should be done simultaneously with the cutting operation. Additionally, in mechanized PCT-work, the weight of inoculum medium which must be carried in the field is less of an obstacle as in motor-manual work.

In recent years, some practical scale experiments for spreading inoculum with clearing saws and devices installed on forest machines have been conducted (Laine et al. 2019, 2020a, b). In motor-manual work, different kinds of nozzle solutions connected to clearing saw blades have been tested (Laine et al. 2020b), and in mechanized PCT-work, a UW40-cleaning head installed on a Tehojätkä mini-harvester (Usewood Forest Tec LTD) and Mense cutting head installed on a normal harvester have been tested (Laine et al. 2019, 2020a). According to these studies, the efficacy of the biological control was not as high as in manual application experiments. In these practical scale experiments, ca. 20–40% of birch stumps died after the fungal treatment. The lack of reliability and accuracy of spreading mechanisms in both motor-manual and mechanized devices were the main reasons for low success rates (Laine et al. 2019, 2020b). In some cases, the stumps were also too tall after mechanized PCT-work, and therefore they continued to grow from branches.

The consumption of inoculum has been high with all studied devices (Laine et al. 2020b). In future, the accuracy of spreading mechanisms should be improved in order to get the consumption of inoculum on an economically sound level. The advantage of the motor-manual and mechanized methods over the manual application methods is the immediate treatment after cutting, which improves the ability of the fungus to infect a stump. In practice, motor-manual and mechanized methods are the only economically feasible solutions. Biological sprout control provides good possibilities for increasing the cost-efficiency of PCT-work by decreasing the number of cases when two-phase PCT-work is needed.

### Potential risks

Intentional spreading of pathogenic organisms is always dangerous, and therefore risk analyses should be conducted carefully prior to such actions on a wide scale (e.g., Hantula et al. 2012). Considering the biocontrol use of *C. purpureum*, the first problem might be the infection of nontarget trees due to the increased production of basidiospores. This possibility was studied in North America by Wall (1991) and in Europe by de Jong et al. (1990a, b) and Vartiamäki et al. (2009b). All these studies suggested that the risk is small, and even the assumption of considerably increased spore load compared to natural levels may be incorrect (de Jong et al. 1996). However, it may be possible that in favorable environmental conditions, control agents could infect freshly wounded non-target trees, although a long distance spread of spores has been shown to be unlikely (de Jong et al. 1990a, b).

The second obvious risk is a possible major change in the genetic composition of *C. purpureum* populations due to biocontrol actions. Therefore, the population genetics of the control fungus should be understood before widespread control actions are conducted. As pointed out above, the populations of *C. purpureum* have been studied in northern Europe and North America, and in both continents the degree of genetic variation is high, but differentiation between populations is small (Becker et al. 2004, 2005; Gosselin et al. 1996, 1999; Hamberg et al. 2018; Ramsfield et al. 1996, 1999; Vartiamäki et al. 2008b). Therefore, either a gene flow occurs naturally over large geographic distances, or the population size of the fungus is so large in both continents that the frequencies of selectively neutral genes change extremely slowly. In terms of biocontrol risks, this can be interpreted so that the use of local genotypes of *C. purpureum* will not lead to introduction of novel genes or genotypes to the natural environment.

However, the case is completely different if *C. purpureum* strains would be transmitted between the continents. Although the strains in Europe and North America belong to the same biological species, they differ considerably in genetic markers (Hamberg et al. 2018). Therefore, introducing these strains between North America and Europe could lead to unexpected consequences as demonstrated by the high degree of damage by the introduction of North American *Heterobasidion irregulare* Garbel. & Otrosina (2010) and its hybridization with the European *H. annosum* (Fr.) Bref. (1888) in Italy (D’Amico et al. 2011; Gonthier and Garbelotto 2011).

Population analyses (Gosselin et al. 1996, 1999; Ramsfield et al. 1999; Vartiamäki et al. 2008b) have also suggested that *C. purpureum* has no asexual (i.e., clonal) reproduction. Therefore, the genes of the biocontrol strain will mix freely with those of the natural population of *C. purpureum* as soon as basidiospores are produced. In this process, the combination of alleles in the obviously efficient isolate to be used will break down by genetic recombination, and the original genotype will not spread outside the treated areas. Therefore, the risk of widespread dispersal of a highly virulent *C. purpureum* clone does not exist.

The unwinding of allele combination is true also, as in the case of the Finnish biocontrol strain R5, which was created by a short breeding program (Hamberg et al. 2015). In that case, however, another question arises: whether the spread of this strain with obviously a high number of virulence alleles increases overall pathogenicity of the naturally spreading *C. purpureum* population. There is no study available regarding this risk, but according to population genetics theory, the extremely large size of *C. purpureum* populations (and thus the vastness of the gene pool) in nature will slow down such a change, if it even occurs. Regardless, the development of genetic diversity of this fungus should be followed over time where its use in the biocontrol of hardwood sprouting becomes a common practice.

### Sprout control in the future

As far as we know, commercially available biocontrol products based on *C. purpureum* do not exist currently, although some products have been registered (see de Jong 2000). Difficulties in getting to market are probably not in the production lines nor in the efficacy of the treatment agent, as the fungus grows well on artificial media and is excellent in controlling the sprouting of the target tree species (see above). Instead, the major dilemma is the lack of cost-efficient treatment protocols for large-scale usage (Laine et al. 2020b).

In Finland, this has led to a self-defeating circle: as there is no cost-efficient mechanized devices available, the possible producer(s) are not willing to register the product in the EU, and as there is no registration, the possible developers of such machinery are not interested in investing in such developments. The need for an efficient sprout control is, however, increasing as the workforce for manual treatments is declining, and therefore the self-defeating circle will probably be broken sooner or later. If so, the final solution could be a lightweight mini-harvester due to the need for carrying large amounts of liquids.

The economic limit—i.e., the challenge to be overcome—of using biological control in forest regeneration sites can be set to the price of the second round of a man-made treatment with a clearing saw, as today two-phase PCT-work is typically conducted in northern Europe. The main issue to be solved will be how to target the biocontrol agent exactly on the stumps of cut hardwoods which are small in diameter, instead of spreading most of it off the target. In order to solve this, both purely mechanical solutions where collecting aligns the stump surfaces with the face of the treatment nozzle and more advanced technology using, e.g., artificial vision and intelligence to recognize and individually treat each stump surface can be imagined. In both cases, the additional time consumption of the treatment should be minimized (Laine et al. 2020b).

As genetic differences between *C. purpureum* on different continents are obvious despite their biological conspecificity (Hamberg et al. 2018), none of the control agents will probably be used globally. However, the low level of population differentiation (Becker et al. 2004, 2005; Gosselin et al. 1996, 1999; Ramsfield et al. 1996, 1999; Vartiamäki et al. 2008b) within continents allows the use of a single strain over large areas in Europe or North America. Also, development of resistance in hardwoods against any of these strains is unlikely due to the high frequency of natural infection. However, if there will be a need or willingness for new control strains, a breeding program is probably more efficient than simple testing of natural strains in order to find high efficacy (Hamberg et al. 2015). It might be even more efficient to develop more virulent strains via genetic modification or gene editing, but we consider it highly unlikely that such strains of already highly efficient plant pathogens would ever be developed.

## Conclusion

A decay fungus, *Chondrostereum purpureum*, is efficient in decaying wood and as a fungal inoculum is a potential option in controlling excessive regrowth of hardwoods from stumps. Sprout control efficacy of *C. purpureum* varies in different tree species, and the growth of fungal hyphae within a stump is also affected by the physiological state of a host tree. Biological sprout control is not sensitive to environmental conditions, and therefore it can be used also during hot and rainy periods and in different soil moisture conditions. However, fungal inoculum should be spread on fresh stump surfaces immediately after cutting. From this point of view, fully mechanized devices capable of carrying the weight of fungal inoculum are optimal options in biological sprout control in the future.

## Data Availability

Not applicable.
